# Multitasking in older adults with type 2 diabetes: A cross-sectional analysis

**DOI:** 10.1371/journal.pone.0186583

**Published:** 2017-10-18

**Authors:** Jason L. Rucker, Joan M. McDowd, Jonathan D. Mahnken, Jeffrey M. Burns, Carla H. Sabus, Amanda J. Britton-Carpenter, Nora B. Utech, Patricia M. Kluding

**Affiliations:** 1 Department of Physical Therapy and Rehabilitation Science, University of Kansas Medical Center, Kansas City, Kansas, United States of America; 2 Department of Psychology, Rockhurst University, Kansas City, Missouri, United States of America; 3 Department of Biostatistics, University of Kansas Medical Center, Kansas City, Kansas, United States of America; 4 Department of Neurology, University of Kansas Medical Center, Kansas City, Kansas, United States of America; University of Florida, UNITED STATES

## Abstract

**Background and purpose:**

Deficits in the ability to multitask contribute to gait abnormalities and falls in many at-risk populations. However, it is unclear whether older adults with type 2 diabetes mellitus (DM) also demonstrate impairments in multitasking. The purpose of this study was to compare multitasking performance in cognitively intact older adults with and without DM and explore its relationship to measures of gait and functional ability.

**Methods:**

We performed a cross-sectional analysis of 40 individuals aged 60 and older with type 2 DM and a matched group of 40 cognitively intact older adults without DM. Multitasking was examined via the ambulatory Walking and Remembering Test (WART) and seated Pursuit Rotor Test (PRT). Self-selected normal and fast walking speed and stride length variability were quantitatively measured, and self-reported functional ability was assessed via the Late Life Function and Disability Index (LLFDI).

**Results:**

Participants with DM walked slower and took more steps off path when multitasking during the WART. No between-group differences in multitasking performance were observed on the PRT. Multitasking performance demonstrated little correlation with gait and functional ability in either group.

**Discussion and conclusions:**

Older adults with DM appear to perform poorly on an ambulatory measure of multitasking. However, we analyzed a relatively small, homogenous sample of older adults with and without type 2 DM and factors such as peripheral neuropathy and the use of multiple comparisons complicate interpretation of the data. Future research should explore the interactions between multitasking and safety, fall risk, and function in this vulnerable population. Clinicians should recognize that an array of factors may contribute to gait and physical dysfunction in older adults with type 2 diabetes, and be prepared to assess and intervene appropriately.

## Introduction

Dividing attention between simultaneous activities in order to multitask is a highly functional human behavior. This complex balancing of attentional resources is often described as an executive function [1] and can be elicited by dual-task paradigms that examine the changes in performance that occur when multiple tasks are undertaken simultaneously [2]. Such dual-task studies suggest that an impaired ability to multitask may influence gait and function in a number of high risk groups [3–5], including the rapidly increasing population of older adults with type 2 diabetes [6, 7].

Characterized by impairments in insulin production and utilization and the resulting dysregulation of glucose levels, diabetes affects approximately 29.1 million individuals, including over one-quarter of all older adults [8]. Type 2 diabetes accounts for up to 95% of this number [8] and, alarmingly, is projected to afflict 1 in every 3 Americans by the year 2050 [9].

There is no question that diabetes can affect the peripheral nervous system [10], and functional deficits in individuals with diabetes are often attributed to peripheral neuropathy. However, gait abnormalities have been observed in individuals with diabetes but no evidence of neuropathy [11, 12], and some have reported that depression and cognition may be nearly as much related to gait and physical function as signs and symptoms of neuropathy in this population [13, 14]. While considerable evidence has linked diabetes to cortical damage in areas linked to neuropsychiatric and executive functions [15], the degree to which neuropsychological and cognitive disturbances influence gait and function in those with diabetes remains largely unknown. Moreover, older adults with diabetes may suffer from an increased fear of falling and loss of balance confidence [16], which could exacerbate gait disturbances regardless of somatosensory loss [17].

Also of interest is evidence that walking while performing a cognitive task may disrupt spatiotemporal gait parameters in individuals with diabetes, including gait speed [6], stride length and velocity [7], stride length variability [7], step length and time [6], and double support time [6, 7]. The fact that these changes have been observed in individuals both with and without neuropathy could potentially reflect a deficit in the cognitive ability required to divide attention between the tasks.

To date, relatively little research has investigated multitasking in older adults with diabetes, or explored whether it may contribute to the widespread incidence of functional deficits observed in this population. The primary hypothesis of this study was that older adults with type 2 diabetes would demonstrate poorer performance on two measures of multitasking, the Walking and Remembering and Pursuit Rotor Tests, when compared to older adults without diabetes matched for age, sex, education, and hypertension status. Secondarily, we explored whether multitasking performance in those with diabetes would be correlated with quantitative measures of gait and self-reported measures of physical function and disability.

## Methods

### Study design and sample

Institutional approval for this cross-sectional study was granted by the University of Kansas Medical Center Human Subjects Committee.

**Fig 1:**
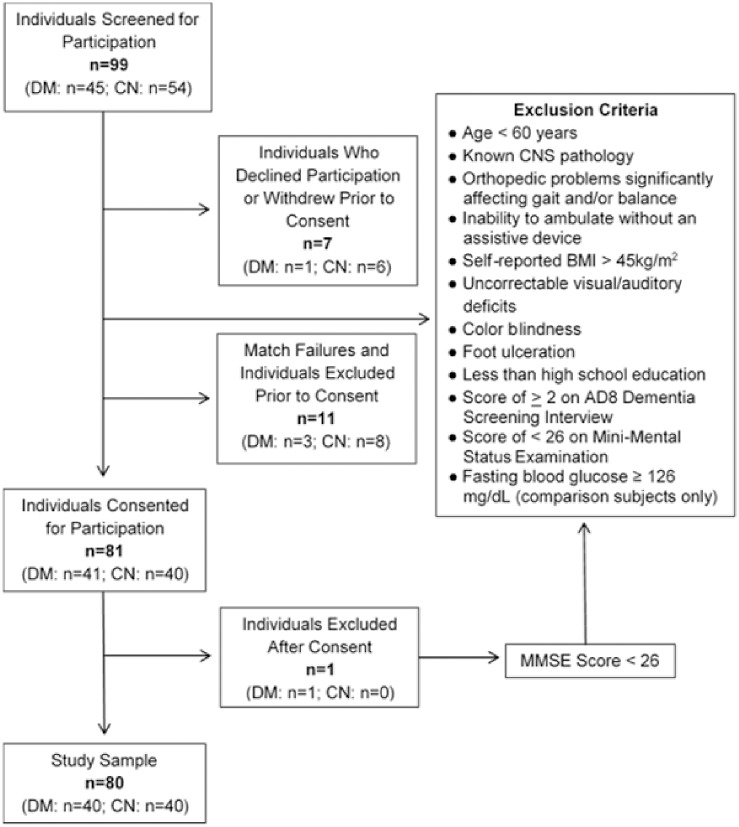
Study flowchart. Flowchart of participants throughout the study. Abbreviations: DM, type 2 diabetes mellitus; CN, comparison; CNS, central nervous system; BMI, body mass index; MMSE, Mini-Mental Status Examination.

Between January, 2011 and April, 2013, a total of 99 individuals aged 60 and older with and without a medical diagnosis of type 2 diabetes were identified through databases maintained by the University of Kansas Medical Center’s Institute for Clinical and Translational Research, Alzheimer’s Disease Center, Center on Aging, and Georgia Holland Laboratory (Fig 1). These individuals were screened for the following exclusion criteria: 1) history of central nervous system pathology, 2) musculoskeletal conditions significantly affecting gait and/or balance, 3) inability to ambulate without an assistive device, 4) body mass index of >45 kg/m2, 5) uncorrectable visual/auditory deficits or color blindness, 6) wounds on the weight bearing surfaces of the feet, 7) less than a high school level of education, and/or 8) cognitive impairment as evidenced by a score of ≥2 on the AD8 Dementia Screening Interview (pre-consent) or a score of <26 on the Mini-Mental Status Examination (post-consent).

Forty participants with diabetes (DM) were individually matched to comparison participants (CN) in terms of age (± 5 years), sex, level of education completed (high school vs. college), and the presence of hypertension. This yielded a total sample of 40 matched subject-pairs. Participants were provided with a $50 stipend upon completing the study.

### Procedures

Following the signature of an institutionally-approved informed consent form, age, height, weight, medical comorbidities, current medications, and number of falls in the past 6 months were recorded. A fasting blood glucose measurement was obtained from each participant (Contour Blood Glucose Monitoring System, Bayer, Tarrytown, NY). Glycosylated hemoglobin (HbA1c) levels were measured for each participant with diabetes (Metrika A1CNow+, Bayer, Tarrytown, NY). Potential comparison participants exhibiting fasting blood glucose levels ≥126 mg/dL were excluded and referred to a physician for further metabolic evaluation. A small snack was made available to each participant once their fasting blood glucose level was established. All participants completed testing in a normal glycemic state, defined as a blood glucose level of between 80 and 250 mg/dL.

After glycemic testing, each participant completed the Mini-Mental Status Examination (MMSE) [18] to assess global cognitive function, Beck’s Depression Inventory-II to assess signs and symptoms of depression [19], and a self-reported assessment of functional mobility, the Late Life Function and Disability Index (LLFDI) [20]. This was followed by two randomly ordered measures of multitasking, the Walking and Remembering and Pursuit Rotor Tests, and quantitative analysis of gait speed and variability. All testing was conducted by the same research personnel in a quiet laboratory setting to minimize distraction.

### Multitasking assessments

1) The Walking and Remembering Test (WART) is a clinical measure of multitasking ability involving the individual (e.g. single-task) and simultaneous (e.g. dual-task) performance of ambulatory and cognitive tasks [21]. This test requires participants to remember a random sequence of numbers while ambulating as quickly as possible along a marked 6.1m long, 19cm wide path, keeping both feet within the lines of the path. Each task component is recorded under single-task (e.g. walking only) and dual-task conditions (e.g. walking while remembering a random series of digits). Participants wore their normal footwear and were not allowed to utilize an assistive device.

Following a demonstration of the test, participants first performed 4 trials of the walking component of the WART under single-task conditions, with instructions to walk as quickly and safely as possible while staying within the path. A step off path was counted if the participant’s foot fell completely outside of the marked pathway. A seated forward digit span test was then administered to determine an appropriate cognitive challenge, defined as the longest random number sequence the individual could correctly recall. The number of correctly recalled digits on the seated forward digit span test reflected single-task cognitive performance, while the average walking speed and number of steps off path across the 4 walking only trials of the WART reflected single-task walking performance.

For dual-task conditions, participants completed 4 trials in which they performed the walking task while simultaneously attempting to remember a random sequence of numbers. To accomplish this, participants stood at the beginning of the WART walking path, and were read a sequence of random numbers whose length was equal to that participant’s maximum seated forward digit span performance. Immediately after the sequence was read, the participant ambulated along the walking path as previously described, while simultaneously attempting to remember the number sequence. Upon reaching the end of the path, the participant was asked to recall as many of the numbers in the sequence as possible in the correct order. The average number of correctly recalled digits, walking speed, and the number of steps off path across the 4 walking and remembering trials of the WART reflected dual-task performance.

2) The Pursuit Rotor Test (PRT) is an internally developed computerized multitasking measure (Digital Electronics and Engineering Core, Biobehavioral Neurosciences and Communication Disorders Center, University of Kansas, Lawrence, KS) in which participants used a trackball mouse (Kensington Technology Group, Redwood Shores, CA) to pursue a target around an elliptical track while performing a verbal fluency task [22].

All participants practiced the tracking task while target speed was adjusted until the average time on target plateaued and oscillated around 80% accuracy. A 1-minute trial was then administered to determine single-task tracking performance. Next, two single-task verbal fluency trials were conducted, in which participants were provided a letter of the alphabet (F and M) and asked to say as many words as possible (excluding proper nouns) beginning with that letter in 1-minute. The average number of words generated during these two trials reflected single-task verbal fluency. Two trials of the multitasking condition followed, in which participants tracked the target for 1-minute while simultaneously completing the verbal fluency task using different letters of comparable frequency (B and L) [23]. A final 1-minute single-task tracking trial completed the test, and the average time on target, error distance, and number of words obtained under single- and dual-task conditions were recorded.

### Gait and functional assessments

1) Quantitative gait analysis was conducted using a GaitMat II analysis system (E.Q., Inc., Chalfont, PA), consisting of a 4-meter long walkway housing 38 rows of 256 pressure sensitive switches connected to a computer analysis system via a USB interface. Participants began walking approximately 2-meters before stepping onto the GaitMat, and were instructed to continue walking to a point approximately 2-meters beyond the GaitMat. Three trials were conducted at a self-selected “normal” walking speed, followed by three trials in which participants were instructed to walk “as quickly and safely as possible”. Participants wore their normal footwear and were not allowed to utilize an assistive device. Gait velocity (in m/s) and stride length variability (expressed as the coefficient of variation: [SD/mean]*100) for both normal and fast walking conditions were averaged across the 3 trials.

2) The Late Life Function and Disability Instrument (LLFDI) is a comprehensive measure of physical function and disability specifically designed for older adult populations [20]. The physical function component consists of 32 items evaluating self-reported difficulty in physical activities involving upper extremity function, lower extremity function, and advanced lower extremity function (e.g. running, etc.). Participants were asked “How much difficulty do you have doing [a particular activity] without the help of someone else and without the use of an assistive device?” and responded “none”, “a little”, “some”, “quite a lot”, or “cannot do.” Overall physical functioning was scored on a scale of 0–100, with higher scores reflecting higher levels of function.

A further 16 items assessed frequency of participation in and ability to perform major life tasks. Participants were asked how often they performed a particular task, and rated the extent to which they felt limited with responses of “not at all,” “a little,” “somewhat,” “a lot,” and “completely”. Frequency and disability indices were also scored on scales of 0–100, with higher scores reflecting higher frequency of participation and lower degree of disability.

### Data analysis

Analyses were performed using SPSS 16.0 for Windows (Chicago, IL). Descriptive statistics were calculated, and distribution and variance examined via scatter and Q-Q plots and assessed with Kolmorgorov-Smirnov and Shapiro-Wilk tests. Normally distributed data are presented as mean (standard deviation) and non-normally distributed data as median (minimum–maximum). One outlying data point was identified in steps off path on the WART; however, analyses conducted with and without this data point were not statistically different and it was not removed from the final analysis [24].

### Power analysis

An appropriate sample size was determined via a power analysis, with differences in multitasking performance identified as the primary outcome of interest. This analysis utilized cognitive-motor multitasking data obtained from a pilot sample of individuals with diabetic peripheral neuropathy [14] and studies of cognitive-motor multitasking in healthy older adults [25]. Based on an estimated effect size of 0.44, this analysis indicated that a sample of 40 participants per group would yield a power of between 74 and 86%, depending upon the balance of discrete and overlapping information provided by the two measures of multitasking.

### Dual-task cost calculation

Multitasking test components were analyzed, in part, as the percent change in performance, or dual-task cost, from single- to dual-task conditions. This was calculated via the following formula:

$$\left|Dual\;task\;Cost\right|=\;\frac{\left(Dual\;task\;performance\;-Single\;task\;performance\right)}{Single\;task\;performance}\;x\;100$$

By convention, a positive dual-task cost reflects a decline in task performance from single- to dual-task conditions (e.g. a 5% decline in walking speed), whereas a negative dual-task cost represents an improvement in performance under dual-task conditions. Individual dual-task costs for each component of the two multitasking tests were averaged to generate a total dual-task cost for each measure. For example, the total dual-task cost for the WART reflected the average of the dual-task costs elicited for digit span recall, walking speed, and steps off of the path.

### Comparisons

Because each participant with diabetes was individually paired to a comparison group participant, between- and within-group differences and 95% confidence intervals were examined via 2-tailed, 1-sample paired t-tests. The Wilcoxon signed-rank test assessed differences in non-normally distributed data. Pearson-product-moment and Spearman’s rank-sum correlations explored the relationships between normally and non-normally distributed variables, respectively. Type I error rate was set at 0.05.

## Results

### Sample characteristics

Table 1 contains the general sample characteristics. Forty older adults with type 2 diabetes (65% female) and 40 paired individuals without diabetes (65% female) participated in the study. Seventy-eight percent of both groups were college educated, with the same percentage reporting a diagnosis of hypertension.

**Table 1:** Sample characteristics^a^.

	Diabetes (n = 40)	Comparison (n = 40)	p-value	95% CI
**Age** **(years)**	72.9 (8.3)	72.9 (7.7)	0.90	-1.1–1.2
**BMI** **(kg/m^2^)**	31.1 (4.7)	26.6 (4.4)	<0.001	2.6–6.4
**FBG** **(mg/dL)**	134.3 (45.9)	92.3 (14.1)	<0.001	25.0–51.3
**MMSE** **(score out of 30)**	28.7 (1.2)	29.2 (1.0)	0.07	-1.0–0.04
**BDI** **(score out of 63)**	4.5 (0–28)	3.0 (0–31)	0.04^b^	-0.4–5.2
**Fall History (%)**^c^	15% (6/40)	5% (2/40)	--	--

Abbreviations: CI, confidence interval; BMI, body mass index; FBG, fasting blood glucose; MMSE, Mini-Mental Status Examination; BDI, Beck’s Depression Inventory-II.

^a^Results are presented as mean (SD) for normally distributed data and median (minimum–maximum) for non-normally distributed data.

^b^Wilcoxon signed-rank test.

^c^Self-reported history of ≥2 falls in past 6 months.

As expected, participants with diabetes exhibited higher fasting blood glucose (p<0.001) and were more obese (p<0.001). Those with diabetes reported more symptoms of depression than comparison participants (mean BDI 6.6±6.1 vs. 4.2±5.7 respectively, p = 0.04), and longer-term glycemic control in the diabetes group was also impaired (mean HbA1c 7.0±1.3%, range 5.4–11.2). Eight participants (20%) in this group reported a diagnosis of peripheral neuropathy, 2 (5%) mild retinopathy, and 1 (2.5%) nephropathy. A total of 6 participants (15%) in the diabetes group and 2 in the comparison group (5%) reported 2 or more falls in the preceding six months. No significant between-group differences were observed in age or global cognitive function.

### Multitasking performance

**Fig 2:**
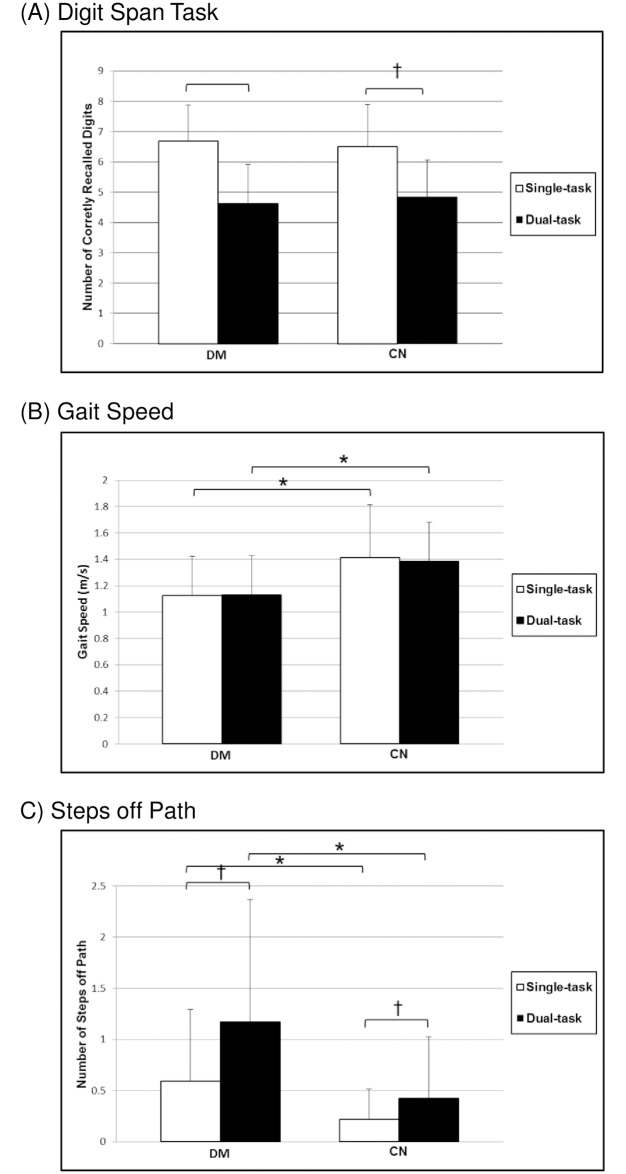
Walking and Remembering task performance. (A) Average number of digits recalled, (B) gait speed, and (C) number of steps off path under single-task (white bars) and dual-task (black bars) conditions on the Walking and Remembering Test. Error bars reflect standard deviation from the mean. Within-group differences in height between single- and dual-task bars reflect the changes in task performance (e.g. dual-task costs) for each task. * Significant between-group difference at p<0.05. † Significant within-group difference at p<0.05.

**Fig 3:**
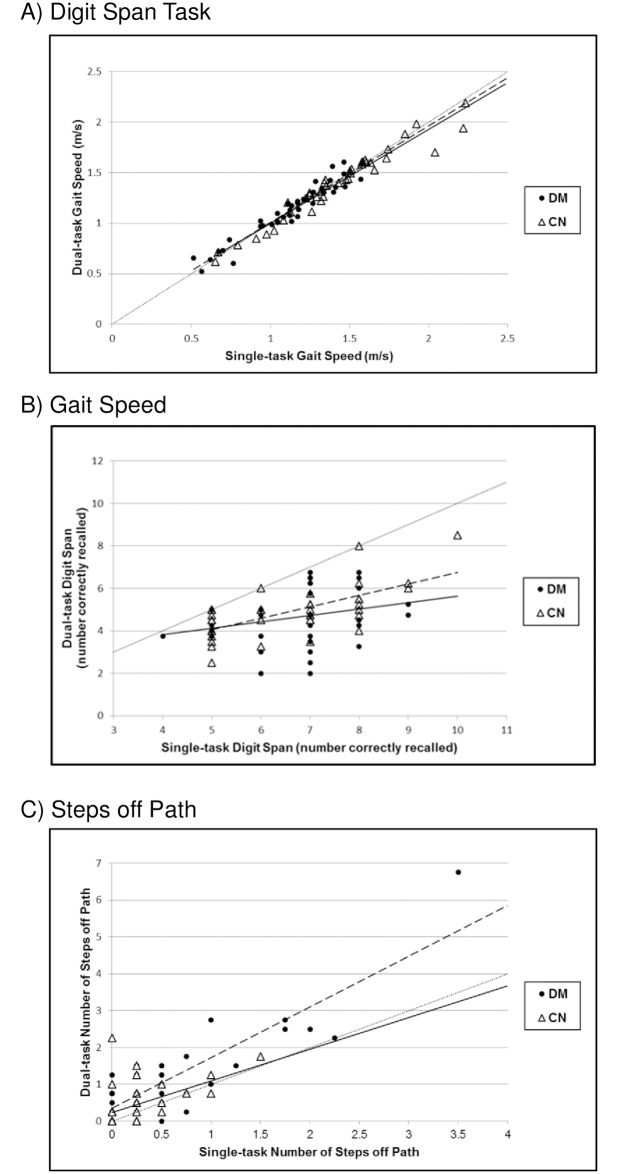
Dual-task costs on the Walking and Remembering Test. (A) Percent changes (e.g. dual-task costs) from single- to dual-task conditions in digit span performance, (B) gait speed, and (C) steps off path on the Walking and Remembering Test. Lightly dotted lines represent no change in performance (dual-task cost) from single- to dual-task conditions. Dashed and solid lines reflect trend lines for the diabetes and comparison groups, respectively. In (A) and (B), points falling below the dotted line reflect poorer cognitive performance and slower walking speed under dual-task conditions. In (C), points falling above the dotted line reflect more steps off path under dual-task conditions.

Results of the Walking and Remembering Test are provided in Table 2 and illustrated in Figs 2 and 3. Pursuit Rotor Test results are provided in Table 2. Consistent with our primary hypothesis, the diabetes group demonstrated poorer overall performance on the WART (p = 0.005); however, we did not observe significant between-group differences in overall PRT performance (p = 0.14).

**Table 2:** Testing results^a^.

	Diabetes (n = 40)	Comparison (n = 40)	p-value	95% CI
**WART Total**^b^ **(% change)**	34.7 (-29.7–186.3)	12.3 (-24.1–171.3)	0.005^d^	0.01–0.40
**WART Recalled Digits** **(% change)**	29.5 (19.4)	24.3 (14.3)	0.21	-0.03–0.13
**WART Walking Time** **(% change)**	-0.01 (0.08)	0.02 (0.05)	0.11	-0.06–0.01
**WART Steps Off Path** **(% change)**	75.0 (-100.0–500.0)	0.0 (-100.0–500.0)	0.008^d^	0.01–1.2
**PRT Total**^c^ **(% change)**	1.1 (12.8)	4.7 (10.1)	0.14	-0.1–0.01
**PRT Verbal Fluency** **(% change)**	-7.3 (25.3)	1.5 (17.7)	0.05	-4.4 –-0.21
**PRT Time on Target** **(% change)**	3.4 (-13.1–34.3)	4.6 (-17.0–22.7)	0.49^d^	-0.04–0.04
**PRT Error Distance** **(% change)**	5.7 (27.4)	7.3 (25.1)	0.80	-0.14–0.11
**Normal Gait Speed** **(m/s)**	1.0 (0.2)^e^	1.1 (0.2)^e^	0.03	-0.18 –-0.01
**Fast Gait Speed** **(m/s)**	1.14 (0.4)^e^	1.6 (0.3)^e^	0.001	-0.33 –-0.09
**Normal Stride Length Variability (%)**	4.3 (0.1–16.1)^e^	4.6 (2.4–15.9)^e^	0.77^d^	-1.1–1.9
**Fast Stride Length Variability (%)**	5.4 (2.5)^e^	4.0 (1.7)^e^	0.02	0.3–2.5
**LLFDI Frequency** **(score out of 100)**	56.3 (6.5)	58.6 (9.7)	0.42	-4.5–1.9
**LLFDI Disability** **(score out of 100)**	79.7 (14.2)	85.3 (13.8)	0.06	-11.3–0.1
**LLFDI Physical Function** **(score out of 100)**	60.6 (9.6)	67.5 (8.2)	<0.001	-10.1 –-3.7

Abbreviations: WART, Walking and Remembering Test; PRT, Pursuit Rotor Test; LLFDI, Late Life Function and Disability Index.

^a^Results are presented as mean (SD) for normally distributed data and median (minimum–maximum) for non-normally distributed data.

^b^WART Total reflects the average of the dual-task costs elicited for digit span recall, walking speed, and steps off of the path.

^c^PRT Total reflects the average of the dual-task costs elicited for verbal fluency, time on target, and error distance.

^d^Wilcoxon signed-rank test.

^e^n = 35.

#### Walking and Remembering Test

Analysis of WART components (Table 2, Figs 2 and 3) revealed that the groups demonstrated similar cognitive performance under single-task conditions (p = 0.54); however, those with diabetes ambulated more slowly (p<0.001) and took more steps off path (p<0.001). Multitasking resulted in a within-group decline in digit recall performance (DM: p<0.001, CN: p<0.001) and an increase in the number of steps off path (DM: p<0.001, CN: p = 0.004) in both groups. No significant changes in gait speed were observed in either group while multitasking (DM: p = 0.79, CN: p = 0.06). The percent changes, or dual-task costs, for cognitive task performance and gait speed were not different between the groups (p = 0.21 and 0.11, respectively); however, those with diabetes exhibited a greater dual-task cost in the number of steps off path (p = 0.008).

In addition, we performed bivariate correlations to explore whether sample characteristics such age, obesity, glycemic control, overall cognitive function, and depression were associated with the changes in WART task performance we observed in subjects with diabetes. Table 3 provides the results of this analysis. Fasting blood glucose demonstrated a weak, inverse correlation to the change in cognitive task performance. Otherwise, only small, non-significant relationships existed between these variables and the dual-task costs associated with digit span recall, walking speed, and steps off path.

**Table 3:** Relationships between sample characteristics and WART task performance in subjects with diabetes.

	Dual-task Cost Digit Span	Dual-task Cost Walking Speed	Dual-task Cost Steps off Path
**Age**	0.16	-0.21	0.08^a^
**BMI**	0.01	0.02	-0.12^a^
**FBG**	-0.32^a^*	-0.02^a^	0.26^a^
**MMSE**	-0.16^a^	0.10^a^	0.19^a^
**BDI**	0.14^a^	-0.01^a^	-0.18^a^

Abbreviations: WART, Walking and Remembering Test; BMI, body mass index; FBG, fasting blood glucose; MMSE, Mini-Mental Status Examination; BDI, Beck’s Depression Inventory-II.

^a^Spearman rank-sum correlation coefficient.

*Significant at p<0.05.

#### Pursuit Rotor Test

Analysis of PRT components (Table 2) revealed that both groups exhibited a similar time on target (p = 0.21) and distance of error (p = 0.11) under single-task conditions, although those with diabetes performed worse on the verbal fluency task (p = 0.03). Multitasking resulted in a decline in the amount of time on target in both groups (DM: p = 0.002, CN: p<0.001), but did not significantly alter either verbal fluency (DM: p = 0.42, CN: p = 0.29) or distance of error (DM: p = 0.39, CN: p = 0.55). There were no between-group differences in the dual-task costs for verbal fluency performance (p = 0.05), time on target during tracking (p = 0.49), or the distance of tracking error (p = 0.80).

### Quantitative gait analysis

Due to equipment malfunction, quantitative gait data could not be obtained for 5 individuals and data for the corresponding pair-matched participants were also removed from the analysis. Data from the 35 remaining subject-pairs is provided in Table 2. Older adults with diabetes ambulated more slowly than comparison subjects at a self-selected “normal” walking pace (p = 0.03), but with a similar degree of stride length variability (p = 0.77). When instructed to walk “as quickly and safely as possible”, those with diabetes demonstrated slower speeds (p = 0.001) and greater stride length variability than comparison participants (p = 0.02).

### Late Life Function and Disability Index

Results of the Late Life Function and Disability Index are provided in Table 2. The groups scored similarly on the frequency (p = 0.42) and disability (p = 0.06) scales of the LLFDI; however those with diabetes scored significantly lower on the physical function component (p<0.001).

### Relationships between multitasking performance and gait and functional ability

Our secondary hypotheses were that multitasking performance would be significantly correlated with quantitative measures of gait and self-reported functional ability. Bivariate correlations explored these relationships separately in the diabetes and comparison groups. Tables 4 and 5 provide the results of this analysis. Overall, the data did not support our hypotheses, revealing only weak, non-significant relationships between total WART and PRT costs and gait speed, gait variability, and LLFDI scores in both groups.

**Table 4:** Multitasking and other correlates of gait and functional ability in subjects with diabetes.

	Normal Gait		Fast Gait		Late Life Function and Disability Index		
	Speed	Variability	Speed	Variability	Frequency	Disability	Function
**Age**	-0.31	0.20^a^	-0.38*	0.27	-0.25	-0.25	-0.35*
**BMI**	-0.05	-0.15^a^	0.07	-0.26	-0.19	-0.16	-0.27
**FBG**	-0.20	0.28^a^	-0.14	0.06	-0.29	-0.46†	-0.22
**WART**	-0.07^a^	0.22^a^	-0.14^a^	0.29^a^	-0.03^a^	-0.03^a^	0.05^a^
**PRT**	0.09^a^	-0.07^a^	0.11	-0.15^a^	0.33^a^	-0.03^a^	-0.01^a^

Abbreviations: BMI, body mass index; FBG, fasting blood glucose; WART, Walking and Remembering Test; PRT, Pursuit Rotor Test.

^a^Spearman rank-sum correlation coefficient.

*Significant at p<0.05.

^†^Significant at p≤0.01.

**Table 5:** Multitasking and other correlates of gait and functional ability in control subjects.

	Normal Gait		Fast Gait		Late Life Function and Disability Index		
	Speed	Variability	Speed	Variability	Frequency	Disability	Function
**Age**	-0.35*	0.29^a^	-0.49†	-0.01	-0.08	-0.08	-0.29
**BMI**	-0.45*	0.17^a^	-0.14	0.44†	-0.02	-0.14	-0.27
**FBG**	-0.25	0.16^a^	-0.12	0.13	0.10	-0.15	-0.27
**WART**	-0.06^a^	0.09^a^	0.12^a^	-0.14^a^	0.11^a^	-0.06^a^	0.22^a^
**PRT**	-0.17^a^	0.19^a^	-0.15^a^	-0.06^a^	0.24^a^	0.08^a^	-0.05^a^

Abbreviations: BMI, body mass index; FBG, fasting blood glucose; WART, Walking and Remembering Test; PRT, Pursuit Rotor Test.

^a^Spearman rank-sum correlation coefficient.

*Significant at p<0.05.

^†^Significant at p≤0.01.

## Discussion

This study is one of relatively few that have attempted to characterize multitasking abilities in older adults with type 2 diabetes. We observed that these individuals performed comparably to their counterparts without diabetes on a seated measure of multitasking involving upper extremity function, the PRT. However, they fared worse on an ambulatory measure of multitasking, the WART. Analysis of the WART revealed that dual-task costs in cognitive performance and walking speed were comparable in both groups, whereas the dual-task cost associated with steps off path more than doubled in participants with diabetes. These findings may suggest that cognitively intact older adults with diabetes do not necessarily demonstrate global, gross impairments in multitasking. However, when walking, they may fail to adopt compensatory strategies in situations in which task demands outstrip attentional and/or functional resources.

Despite these implications, the multitasking changes we observed appeared to be largely sub-clinical, and we found little correlation between our multitasking assessments and measures of gait and functional ability. This may be due to the fact our sample was restricted to relatively high-functioning older adults who were free from overt cognitive dysfunction. This is important because several authors have found that disturbances in cognitive and executive function are more predictive of dual-task and gait impairments than non-physiological risk factors such as diabetes status [26, 27].

Although we chose to exclude individuals with significant cognitive impairments from our study, both aging [28] and diabetes [15] are known to confer an elevated risk of cognitive impairment and Alzheimer’s disease. It is possible that we did not observe more pervasive differences in multitasking performance or associations between multitasking performance, gait, and functional ability because these are more strongly mediated by cognitive deficits than by risk factors such as diabetes. It is also possible that our characterization of diabetes as a dichotomized risk factor (e.g. diabetes vs. no diabetes) masked the potentially complex relationships between physiological aspects of metabolic function, multitasking performance, gait, and physical function.

It is also important to note that this study was not powered to fully address the relationships between dual-task performance and gait or functional abilities, and our measures of gait and functional measures were limited in scope. Other gait parameters and functional outcomes have been associated with multitasking, falls, and functional deficits, and more extensive assessments of gait and function may better elucidate the relationships between these variables.

To date, few other investigations have compared multitasking abilities in older adults with and without diabetes. Roman de Mettelinge et al. [7] administered dual-task measures of walking while performing serial subtraction by 3 and walking while reciting animal names to older individuals with diabetes and peripheral neuropathy, individuals with diabetes but no neuropathy, and age- and sex-matched comparison participants. They found that multitasking adversely affected parameters of gait speed and variability in both groups with diabetes when compared to controls. These changes were not different between those with and without neuropathy, but did appear to be magnified in individuals exhibiting poorer cognitive and executive function.

Although we did not examine differences between individuals with and without neuropathy, our study also found that participants with diabetes walked slower and with more steps off path than comparison participants under both single- and dual-task conditions. However, we did not observe a change in walking speed while multitasking. Rather, our participants tended to maintain their speed, but took more steps off path. While a corresponding observation was also made in the comparison group, the magnitude of this change (e.g. the dual-task cost) was significantly greater in those with diabetes.

This may have been due to the task characteristics of the WART, which essentially encourage participants to perform the walking task quickly so as to minimize the amount of time needed for the remembering task [21]. It may also be that the subtraction and animal naming tasks utilized by Roman de Mettelinge and colleagues [7] were more difficult for participants with diabetes, potentially due to differences in educational level. Consequently, these individuals may have allocated attention to the cognitive task that comparison participants could instead delegate to the walking task. Our study addressed this task difficulty limitation by using an individually prescribed cognitive task in participants pair-matched for educational level. Notably, we found no between-group differences in digit span performance under either single- or dual-task conditions, but did observe significant within-group declines in cognitive task performance in both groups. This would seem to suggest that the remembering task employed by the WART was similarly challenging for both groups and of sufficient difficulty to elicit dual-task interference.

Another possible difference lies in the fact that these authors instructed their participants to “concentrate equally on walking and the cognitive task”. We did not provide participants with explicit instructions for task prioritization. Rather, we provided only implicit instruction to perform the WART “as quickly and safely as possible” while staying within the lines of the path, as this seemed more reflective of a real-world environment in which task priorities are often not explicit or pre-established.

A number of factors limit interpretation of our data. First, the sample was of modest size and consisted largely of Caucasian participants of middle to upper socioeconomic status. In addition, we did not correct for the multiple comparisons employed in our study, and did not specifically control for comorbid conditions such as depression or obesity. Another limitation is the inclusion of individuals both with and without peripheral neuropathy. Somatosensory loss undoubtedly impacts gait and function; however, neuropathy is notoriously difficult to diagnose and quantify. In order to obtain an adequate sample of individuals with diabetes, as many as half of whom could be expected to exhibit some symptoms of neuropathy, we opted to record whether individuals had been diagnosed with neuropathy but did not exclude them.

This complicates interpretation, but it is worth noting that those who were known to have neuropathy (n = 8) appeared to perform in much the same way as those who did not report neuropathy. Rather than slowing down, those with neuropathy actually increased their walking speed slightly when multitasking on the WART–at an apparent cost of nearly twice the number of steps off path. Interestingly, we observed broadly similar trends in subjects who reported a history of 2 or more falls in the preceding 6 months (n = 8), and those whose BDI scores fell into the upper quartile (BDI score ≥9, n = 16). However, any conclusions that can be drawn from these observations are very limited, and further research is needed to explore how somatosensation, neuropsychiatric and cognitive functions, and task demands interact to influence task prioritization and multitasking during ambulation in these at-risk populations.

The implications of this study should also be considered in the context of the strengths and limitations of the WART. We selected this measure, in part, because the WART employs a cognitive task whose difficulty is individualized and does not rely on verbal or mathematical abilities that may be subtly biased by educational level. This was of particular concern due to the high prevalence of diabetes among those of lower socioeconomic status. The WART also allowed us to examine the trade-offs between cognitive performance, gait speed, and balance. This consideration was especially important because “slowing down”–usually interpreted as an adverse effect of multitasking–could potentially reflect an advantageous compensation in a population at high risk for somatosensory deficits and falls.

However, it should be noted that the neither the WART nor the PRT have been specifically validated in those with diabetes, and the changes we observed on the WART cannot be directly attributed to diabetes. Nor have falls or functional deficits been specifically linked any of the changes we observed on the WART. Nonetheless, it seems plausible that the phenomena we observed could contribute to fall risk in older adults with diabetes, and further research should investigate how cognitive function and multitasking abilities influence gait, physical function, and fall risk in this population.

Despite these limitations, the results of our study are consistent with a growing body of evidence indicating that dual-task performance may provide clinicians with an attractive target for assessment and intervention. Measures such as the WART, cognitive Timed Up and Go [25], and Walking While Talking [29] tests are easily administered and may reveal important information about an individual’s ability to safely coordinate simultaneous cognitive and ambulatory tasks. Additionally, several researchers have reported that dual-task training may help improve the ability to multitask while walking in older individuals with balance deficits [30] and in those with dementia [31]. Our findings do not directly support the efficacy of these strategies for identifying and reducing fall risk or functional deficits in older adults with diabetes. However, they should prompt clinicians to assess whether safety and function are appropriately balanced during ambulatory multitasking, and consider treatment and educational strategies that may promote appropriate functional adaptations in this high-risk population.

## Conclusions

In summary, this investigation provides evidence that older adults with type 2 diabetes perform poorly when multitasking while walking. Although these changes did not appear to exert a substantial influence on gait mechanics or functional abilities under normal circumstances, it remains unclear whether they may affect safety in more challenging situations. Clinicians should recognize that widely varying factors may contribute to gait and physical dysfunction in older adults with type 2 diabetes, and be prepared to assess and intervene appropriately.

## Supporting information

S1 File Study dataset. (XLSX)

S2 File STROBE checklist. (PDF)
